# The stability of life satisfaction in a 15-year follow-up of adult Finns healthy at baseline

**DOI:** 10.1186/1471-244X-5-4

**Published:** 2005-01-18

**Authors:** Heli Koivumaa-Honkanen, Jaakko Kaprio, Risto J Honkanen, Heimo Viinamäki, Markku Koskenvuo

**Affiliations:** 1 Department of Psychiatry, University of Kuopio, Kuopio, Finland. Department of Psychiatry, Kuopio University Hospital, Kuopio, Finland; 2 Finnish Twin Cohort Study, Department of Public Health, University of Helsinki, Finland; 3 Department of Mental Health and Alcohol Research, National Public Health Institute, Helsinki, Finland; 4 Research Institute of Public Health, University of Kuopio, Kuopio, Finland; 5 Department of Public Health; University of Turku, Turku, Finland

## Abstract

**Background:**

While physical health has improved considerably over recent decades in Finland, the disease burden of mental health, especially that of depression, has become increasingly demanding. However, we lack long-term data on the natural course of subjective well-being in the general population. The aim of this study was to investigate the long-term course of self-reported life satisfaction.

**Methods:**

This was a 15-year prospective cohort study on a nationwide sample of adult Finnish twins (N = 9679), aged 18–45 and healthy at baseline, who responded to postal questionnaires in 1975, 1981 and 1990 including a 4-item life satisfaction scale (happiness/easiness/interest in life and feelings of loneliness). Life satisfaction score (range: 4–20) was classified into three categories: satisfied (4–6), intermediate (7–11) and dissatisfied group (12–20). The associations between life satisfaction scores during the follow-up were studied with linear/logistic regression.

**Results:**

Moderate stability and only a slight effect of age or birth-cohort on mean life satisfaction score (LS) were detected. In 1990, 56% of all and 31% of the dissatisfied remained in the same LS category as at baseline. Only 5.9% of the study subjects changed from being satisfied to dissatisfied or vice versa. Correlations between continuous scores (1975, 1981 and 1990) were 0.3–0.4. Baseline dissatisfaction (compared to satisfaction) predicted dissatisfaction in 1981 (OR = 10.4; 95%CI 8.3–13.1) and 1990 (5.6; 4.6–6.8). Multiple adjustments decreased the risk only slightly.

**Conclusions:**

Life satisfaction in adult Finns was moderately stable during 15 years. Among an identifiable group (i.e. the dissatisfied) life dissatisfaction may become persistent, which places them at a greater risk of adverse health outcomes.

## Background

While physical health has improved considerably during recent decades in Finland, the disease burden of mental health – especially that of depression – has become increasingly demanding for the health care services and society. When the global disease burden – including both fatal and non-fatal outcomes – has been assessed, major depression has been shown to be one of its leading causes [1]. However, not only diagnosed depression but also incomplete recovery from depression and subthreshold depressive symptoms have adverse consequences and chronic courses [2-6]. In general, poor mental health affects somatic health and subjective well-being, but poor subjective well-being might also develop into mental disorder and create a loss of functional capacity, if not a sign of an undiagnosed mental disorder already at baseline.

Life satisfaction and happiness are some of the concepts that have previously been viewed as indicators of subjective well-being [7,8]. Life dissatisfaction, even reported by seemingly healthy subjects, is associated with several indicators of poor health or health risk factors, but especially with depressive symptoms [9-11]. Longitudinally, it predicts poor health outcomes such as morbidity, mortality and premature work disability – due to both somatic as well as psychiatric causes – among the healthy but dissatisfied subjects [9,11-15]. When dissatisfaction is repeatedly reported over years the risk of a poor health outcome increases even more [13-15]. Due to these adverse health outcomes and shortened life expectancy among the identifiable group from the healthy general population (i.e. the dissatisfied), more attention should be paid to the natural course of life dissatisfaction in the general population.

However, good mental health is also an area that should be studied in psychiatry. It is something more than the absence of symptoms. It is a mental state that is objectively desirable, indicating for example maturity, emotional and social intelligence, resilience and subjective well-being according to Vaillant [16]. While the possibilities to directly assess mental health at the level of the general population are limited, subjective well-being at the population level can be measured.

Subjective well-being has mainly been investigated in cross-sectional settings and among the elderly, while follow-ups may have been brief and data from the general population have been sparse. Even if subjective well-being in general population has been suggested to be quite stable [9,17-23], it has also been pointed out that the apparent stability should not be due to the insensitivity of measurements to change or due to fact that most people report satisfaction with life [17,20]. However, this is not the case with psychiatric patients and life satisfaction, among whom life satisfaction has been shown to be lower than in any other patient group [9] and to improve markedly concurrently with their recovery from depression [10].

Mental health policy plays an increasingly recognized role in society, but it needs both epidemiological data as well as experts on the field of mental health to monitor the populations and trends [24]. Thus, in psychiatry, we need information on the natural long-term course of subjective well-being in the general population. This study aimed to examine the long-term course of life satisfaction in healthy adults and to determine how strongly self-reported life dissatisfaction predicts future life dissatisfaction.

## Methods

This prospective cohort study with a follow-up from 1975 to 1990 was based on the Finnish Twin Cohort, a nationwide sample of all Finnish same-sex twin pairs born before 1958 with both members alive in 1975. A baseline health questionnaire was sent in 1975 to twin candidates [25]. The follow-up questionnaires in 1981 and 1990 were sent only to verified twins. Furthermore, the 1990 questionnaire was sent only to twins from pairs born in 1930–1957 with both co-twins alive and residing in Finland. The overall response rates to the questionnaires were 89% in 1975, 84% in 1981 and 77% in 1990. The study procedure has been presented in detail elsewhere [12,25].

The questionnaires included a four-question scale for life satisfaction, which was modified from a questionnaire developed for measuring the quality of life for research purposes in Nordic countries [26]. It has been used among all adult age-groups [9,12] as well as among psychiatric patients [9,10,27]. The study subjects were asked to rate aspects of life satisfaction: interest in life, happiness, ease of living and loneliness (very interesting/happy/easy/not at all lonely = 1, fairly interesting/happy/easy = 2, fairly boring/unhappy/hard/lonely = 4, very boring/unhappy/hard/lonely = 5).

Missing data and the response 'cannot say' were scored as 3. If three or four items were missing, the sum score was recorded as 'missing'. Thus, the total score (LS) ranged from 4–20, with **increasing scores indicating a decrease in life satisfaction**. On the basis of the distribution of the sum score (LS), subjects were categorized into the satisfied (LS:4–6), the intermediate group (LS:7–11) and the dissatisfied (LS:12–20) [12]. The intermediate group consisted of those with an LS score within one standard deviation from the mean [9]. At baseline, responses to all four items were provided by 95.8% (N = 22,416) and at least two items, enabling LS to be calculated, by 99.2% (N = 23,212) of all respondents aged 18–45 years. Thus, one or two missing values were recoded as '3' values for 3.4% of subjects.

The criteria for inclusion in the present study were the availability of baseline life satisfaction data, an age of 18–45 years on 1 January 1976 and being a twin (N = 19,973), since only twins were eligible to receive follow-up questionnaires, as well as being healthy at baseline (N = 16,496, see below for criteria). Moreover, the questionnaire was sent in 1990 only to those whose twin partner was alive. Thus, from these eligible subjects, study subjects were those with all three life satisfaction scores available (N = 9679). They consisted of 4466 (46.1%) male and 5213 (53.9%) female twins (Table 1). The mean age (SD) at baseline was 28.8 years (7.5) for men and 28.1 years (7.6) for women. Those subjects who had incomplete follow-up data (N = 6817) were compared with study subjects. Their life satisfaction data was available as follows: 1) LS 1975 and 1981 (n = 4930); 2) LS 1975 and 1990 (n = 385); 3) LS 1975 only (n = 1502).

**Table 1:** Baseline characteristics of the study subjects and those with incomplete follow-up data on life satisfaction*.

	Study subjects		Subjects with incomplete follow-up		p-value
Baseline characteristics	N (9679)	column %	N (6817)	column %	
Sex					<0.001^1)^
Men	4466	46.1	3932	57.7	
Women	5213	53.9	2885	42.3	
					
Age-group					<0.001^2)^
18 – 25	4121	42.6	3100	45.5	
16 – 35	3513	36.3	2431	35.6	
36 – 45	2045	21.1	1286	18.9	
					
Social class					<0.001^3)^
Upper	565	5.8	361	5.3	
Intermediate	2695	27.8	2121	31.1	
Lower	6419	66.4	4335	63.6	
					
Marital status					<0.001^4)^
Cohabiting	5386	55.7	3443	50.6	
Living alone	4291	44.3	3366	49.4	
					
Smoking cigarettes daily					<0.001^5)^
Non-smoker	6698	69.3	3991	58.7	
1 – 19	2344	24.2	2976	30.5	
> 19	627	6.5	737	10.8	
					
Pure alcohol g/month					<0.001^6)^
None	1361	14.1	807	11.9	
1 – 99	4077	42.2	2334	34.3	
100 – 399	2626	27.1	2076	30.5	
400 – 799	1083	11.2	939	13.8	
≥ 800	524	5.4	648	9.3	
					
Physical activity/month					<0.01^7)^
< 1	1138	12.2	888	13.7	
1 – 5	4585	49.3	3045	46.9	
≥ 6	3577	38.5	2563	39.4	
					
Life satisfaction in 1975					<0.001^8)^
4 – 6	2214	22.9	1370	20.1	
7 – 11	6239	64.4	4303	63.1	
12 – 20	1226	12.7	1144	16.8	

* Life satisfaction data not available from 1981 and/or 1990.

^1) ^F(1, 10007) = 165; ^2) ^F(2, 10006) = 7.02; ^3) ^F(2, 10006) = 9.17; ^4) ^F(1, 10002) = 36.1; ^5) ^F(2, 9995) = 94.0; ^6) ^F(4, 9995) = 45.0; ^7) ^F(2, 9786) = 5.50; ^8) ^F(2, 10006) = 29.0

The criteria for baseline health were based on a health questionnaire (Q) and three nationwide registries: the Hospital Discharge Registry (H), the Registry of Specially Refunded Medication (M) and the Cancer Registry (C). Thus, those with symptoms or diseases covering cardiovascular disease, diabetes, chronic obstructive pulmonary disease or malignant cancer, those who used medications for 37 selected chronic somatic or psychiatric diseases, as well as those who were on a work disability pension due to any cause or had an inpatient admission between 1972 and April 1976, were excluded [12]. The specific exclusion criteria for psychiatric disorders covered work disability (Q), inpatient treatment due to psychiatric causes (ICD-8: 290–309) (H), the right to free medication for psychosis before 1977 (M) and use of hypnotics/tranquilizers for more than 10 days in the preceding year (Q).

It has previously been reported that 4-item life satisfaction is associated with a lower age, female sex, cohabiting, an upper social class, non-smoking, lower alcohol consumption and physical activity [9,12,13]. Thus, the multivariate model included baseline variables such as age (18–24/25–34/35–45), sex, marital status (married or cohabiting/single, divorced or widowed), social class (lower/intermediate/upper group), physical activity (at least 30 minutes of exercise < 1/1–5/ ≥ 6 times a month), current smoking status (non-smoker/1–19/ > 19 cigarettes daily) and alcohol consumption (none/1–99/100–399/400–799/ ≥ 800 g pure alcohol/month) [12]. The upper social class consisted of those with at least 13 years of education and sedentary work, while the lower social class consisted of those with less than 10 years of education and work involving at least standing and walking.

Data analysis was carried out using STATA (version 7.0). Since a study subject could be an age- and sex-matched twin sibling of another study subject, not all the observations were necessarily independent. Therefore, correct standard errors were computed by treating each pair of twins as a single unit (i.e. cluster sampling). The statistical significance of differences was tested by estimates of means (SVYMEAN and SVYLC procedure) for continuous variables and by the chi-squared test for categorical variables (SVYTAB procedure), corrected for clustered data and converted into F-statistics. The stability of life satisfaction over time was examined by computing Pearsonian correlation coefficients between continuous variables. To study how former life dissatisfaction predicts later life dissatisfaction, linear and logistic regression for clustered data was used.

## Results

The baseline characteristics of the study population and those whose follow-up data on life satisfaction was not available at all three data collection times is presented in Table 1. Study subjects were more often women, cohabiting, non-smokers and used less alcohol than those whose follow-up data on life satisfaction was incomplete. There were also slight differences in social class and physical activity. Furthermore, study subjects were somewhat more satisfied (mean LS 8.23; 95%CI 8.18–8.29 vs. LS 8.61; 8.54–8.68) and slightly older (mean age 28.4; 28.2–28.6 vs. 27.9; 27.7–28.1) than those with incomplete follow-up data (Table 1).

In the study population with complete follow-up data on life satisfaction, no marked differences were observed in mean LS scores between age or gender groups or measurement times (Table 2). There was only a slight decrease in mean life satisfaction during the follow-up of 15 years. The main decrease took place in women during 1981–1990. When birth cohorts were studied, only those born during 1940–49 showed a trend of decreasing satisfaction throughout the follow-up, regardless of gender, but they were also the most satisfied group at baseline, being then 26–35 years of age. Young men aged 18–25 at baseline were and remained the most dissatisfied group throughout the follow-up.

**Table 2:** Mean (95% CI) life satisfaction (LS) according to sex, birth cohort and current age among 9679 Finnish adults in 1975, 1981 and 1990.

Subjects	(n)	LS 1975	(n)	LS 1981	(n)	LS 1990
All	(9679)	8.23 (8.18 – 8.29)	(9679)	8.26 (8.20 – 8.31)	(9679)	8.35 (8.29 – 8.41)
Men	(4466)	8.30 (8.22 – 8.38)	(4466)	8.33 (8.25 – 8.41)	(4466)	8.38 (8.30 – 8.46)
Women	(5213)	8.18 (8.10 – 8.25)	(5213)	8.19 (8.12 – 8.27)	(5213)	8.32 (8.24 – 8.40)

Birth cohorts, all						
1950 – 57	(4121)	8.46 (8.36 – 8.55)	(4121)	8.31 (8.23 – 8.40)	(4121)	8.45 (8.36 – 8.54)
1940 – 49	(3513)	7.98 (7.90 – 8.07)	(3513)	8.13 (8.05 – 8.22)	(3513)	8.32 (8.22 – 8.41)
1930 – 39	(2045)	8.22 (8.11 – 8.32)	(2045)	8.35 (8.23 – 8.46)	(2045)	8.19 (8.08 – 8.31)

Current age, all						
18 – 23	(3254)	8.50 (8.40 – 8.61)	-	-	-	-
24 – 32	(3608)	8.04 (7.96 – 8.13)	(4520)	8.28 (8.20 – 8.36)	-	-
33 – 45	(2817)	8.17 (8.08 – 8.26)	(4101)	8.19 (8.11 – 8.27)	(6236)	8.39 (8.31 – 8.46)
46 – 51			(1058)	8.39 (8.23 – 8.55)	(1679)	8.37 (8.23 – 8.50)
52 – 60			-	-	(1764)	8.18 -(8.06 – 8.30)

In terms of 3-category LS scores (Table 3), more than half (56%) of the study subjects in 1990 scored in the same category as they did in 1975, but the relationship was less stable for the satisfied (36%) and the dissatisfied (31%) than the intermediate group (69%). However, only 5.9% of the study subjects were satisfied (LS 4–6) at one of the three data collection times but dissatisfied (LS12-20) at one of the other time points.

**Table 3:** The distribution of study subjects according to their self-reported life satisfaction (LS*) in 1975 and 1990.

	LS 1990							
	Satisfied		Intermediate		Dissatisfied		TOTAL	
	N	row %	N	row %	N	row %	N	row %
LS 1975								
Satisfied	785	35.5	1266	57.2	163	7.4	2214	100
Intermediate	1088	17.4	4293	68.8	858	13.8	6239	100
Dissatisfied	122	10.0	728	59.4	376	30.7	1226	100

TOTAL	1995	20.6	6287	65.0	1397	14.4	9679	100

* Life satisfaction score: satisfied (LS 4–6); intermediate (LS 7–11); dissatisfied (LS 12–20).

The correlation was 0.30 between continuous life satisfaction scores in 1975 and 1990, 0.38 between 1975 and 1981 scores and 0.40 between 1981 and 1990 scores. These coefficients were similar for men and women, but lowest among those aged 18–25 (0.26, 0.34 and 0.39, respectively) and highest among those aged 36–45 (0.36, 0.47 and 0.42, respectively). For the total study population the annual auto-correlation was estimated as 0.92 during 1975–90.

Baseline life dissatisfaction predicted future life dissatisfaction (Table 4). This was also true after adjusting for all the covariates as well as when the categories of each covariate were separately investigated. The same pattern was shown both with categorical and continuous life satisfaction scores. The predictive ability was expectedly stronger for the shorter follow-up (1975–1981) than for the total follow-up (1975–1990). When the odds ratios were compared with those which could be calculated for the subjects with incomplete life satisfaction follow-up data, no significant differences were found.

**Table 4:** Prediction of future dissatisfaction (LS 12–20*) according to baseline dissatisfaction. Risk (OR with 95%CI) for the dissatisfied (LS 12–20) compared to the satisfied (LS 4–6) at baseline.

		COMPARISON OF LS SCORES BETWEEN			
		1975 & 1981		1975 & 1990	
Subjects	(n)	OR	(95 % CI)	OR	(95 % CI)
All †	9679	10.42	(8.28 – 13.10)	5.56	(4.55 – 6.80)
Adjusted ‡	9283	9.79	(7.68 – 12.47)	5.23	(4.24 – 6.46)
Men †	4466	14.81	(10.19 – 21.51)	6.11	(4.47 – 8.35)
Adjusted ‡	4298	12.86	(8.74 – 18.91)	5.62	(4.05 – 7.80)
Women †	5213	7.93	(5.89 – 10.68)	5.29	(4.06 – 6.90)
Adjusted ‡	4985	7.94	(5.78 – 10.92)	4.98	(3.78 – 6.56)

* Risk of future dissatisfaction : LS 12–20 vs. LS 4–12

† Adjusted for age

‡ Adjusted simultaneously for age, sex, marital status, social class, alcohol consumption, current smoking and physical activity (cf. method section).

## Discussion

Life satisfaction was moderately stable in healthy adult Finns during a 15 year period. Age or the birth-cohort had only a slight effect on mean life satisfaction. One third of those dissatisfied at baseline remained the same after the 15-year follow-up. The ability of baseline life satisfaction to predict future life satisfaction was strong, but decreased during the follow-up period, which is a trend that has also been suggested previously [23].

Previous studies have indicated that depression and depressive symptoms may have a chronic course [2-6]. On the other hand, in non-patient samples subjective well-being has also been suggested to be stable [17-22]. Concomitant anxiety or personality traits might play a role in this [28-32]. However, our results concerning the possible chronic course of life dissatisfaction are now based on a very long follow-up and a large sample of adults who reported or were found to have no indication of sickness at baseline. Although regression towards the mean in life satisfaction score was shown in the follow-up and greater instability among the dissatisfied and the satisfied than in the intermediate group, a complete shift from one extreme to another was rare.

In Finland the number of new work disability pensions due to depression has strongly increased [33]. However, at the population level, subjective well-being seems not to have decreased correspondingly according to a comparison of two separate cross-sectional national surveys in 1980 and 2000 using the 12-item General Health Questionnaire [34]. Our cohort study with the 4-item life satisfaction scale measured three times during a 15-year follow-up on the same population strengthens these findings. On the other hand, during these years the physical health of the Finnish population has improved in many objectively assessed ways [34], but an improvement has not been seen in mental health indicators or in life satisfaction at population level. Thus, objectively assessed better somatic health or strong national economic growth (an increase of 48% in inflation-adjusted gross national income per capita from 1975 to 1990), which has also taken place during these years, seems not to guarantee better subjective well-being in a population that is globally speaking already quite well-off. This kind of trend has also been suggested previously [16,32,35]. On the contrary, the mean level of subjective well-being, which was previously sufficient to maintain work ability, seems not to meet the requirements of today's working life.

Our results with respect to predictions seemed not to be overestimations. Those subjects whose dissatisfaction might have led to the most adverse result, i.e. death, were excluded from our study. The response to follow-up surveys was somewhat lower among the dissatisfied, but the observed risks among the study subjects did not differ statistically significantly from those obtained from the subjects with incomplete follow-up data. Similarly, when adjusting for follow-up health behavior instead of baseline health behavior variables, these predictions strengthened slightly, but not significantly.

The 4-item life satisfaction scale is easily administered and well accepted. Its sum score was available for 96% (with imputed scores for 99%) of all respondents at baseline [12,13]. This might be due to the low number of items and its ability to tap the positive pole of subjective well-being [7], even if its sum score is also strongly associated with scores obtained by the 21-item Beck Depression Inventory [10,11,13,36]. In the general population, life dissatisfaction predicts both fatal and non-fatal poor long-term health outcomes [11-15]. Thus, it is worthwhile to assess subjective well-being. In general, if only poor subjective well-being is detected, our long follow-up suggests that there seems to be time to intervene.

To prevent a process from leading to more adverse outcomes, subjects should acknowledge their situation and use their own personal resources, if available. According to a panel of experts, the concept of mental health can be regarded as a developmental process providing an individual or a group with the necessary resources to cope with the demands of life without the simultaneous appearance of negatively experienced moods of longer duration [37]. Society and health care services should support the growth of personal resources and start to intervene when these resources are inadequate and poor subjective well-being persists. In psychiatry, however, according to Vaillant, "since primary prevention is clearly superior to treating disease once it has occurred, we need to study also individuals with positive mental health the way that agronomists study wheat that is resistant to drought and blight" [16].

Our large nationwide sample with a high response rate and a long follow-up period enabled an examination of the long-term course of life satisfaction. The exclusion criteria for baseline health disorders were comprehensive and based on both self-reports and several national registries with high coverage and validity [38-41]. Although these analyses were performed on individuals drawn from a twin cohort, the results should be applicable to the general population. Being a twin does not affect the predictive ability of life satisfaction for mortality or suicide [12,13], and there is at most only a modest contribution of genetics to inter-individual differences in life satisfaction [42]. However, the potential influence of twinship was taken into account in the statistical analysis. The arbitrary exclusion of those twins who did not have a living twin partner in 1990, required in the composition of the Twin Finnish Cohort data, enabled us to control for the loss of a twin sibling.

## Conclusions

Life satisfaction among healthy adult Finns was moderately stable in a 15-year follow-up. Since the dissatisfied, one third of whom consistently rated themselves as dissatisfied, can be identified from the general population, and since dissatisfaction places them at a risk, and repeatedly reported dissatisfaction at even greater risk, of adverse health outcomes, assessing subjective well-being should be encouraged both in surveys and in clinical practice in order to identify those in need of further evaluation of their mental health.

## Competing interest

The author(s) declare that they have no competing interest.

## Authors' contributions

KJ participated in composing the Finnish Twin Cohort data, in planning, commenting on, revising and approving the final manuscript.

KM participated in composing the Finnish Twin Cohort data, in planning, commenting on, revising and approving the final manuscript.

HR participated in planning, commenting on, revising and approving the final manuscript.

HV participated in planning, commenting on, revising and approving the final manuscript.

KHH planned the study, performed the statistical analyses and was the main author.

## Pre-publication history

The pre-publication history for this paper can be accessed here:


